# Biocompatibility and Antibacterial Activity of Eugenol and Copaiba Essential Oil-Based Emulsions Loaded on Cotton Textile Materials

**DOI:** 10.3390/polym16162367

**Published:** 2024-08-21

**Authors:** Laura Chirilă, Miruna S. Stan, Ionela C. Voinea, Alina Popescu, Alexandra-Gabriela Ene, Maricel Danu, Constanța Ibănescu, Mihaela-Cristina Lite

**Affiliations:** 1 National Research and Development Institute for Textiles and Leather—INCDTP, Lucrețiu Pătrășcanu 16, 030508 Bucharest, Romania; laura.chirila@incdtp.ro (L.C.); alina.popescu@incdtp.ro (A.P.); alexandra.ene@incdtp.ro (A.-G.E.); cristina.lite@incdtp.ro (M.-C.L.); 2 Department of Biochemistry and Molecular Biology, Faculty of Biology, University of Bucharest, 91–95 Splaiul Independentei, 050095 Bucharest, Romania; ionela-cristina.voinea@bio.unibuc.ro; 3 “Cristofor Simionescu” Faculty of Chemical Engineering and Environmental Protection, “Gheorghe Asachi” Technical University of Iasi, 73 Prof. Dr. Docent D. Mangeron Blvd, 700050 Iasi, Romania; constanta.ibanescu@gmail.com; 4 “Petru Poni” Institute of Macromolecular Chemistry, 41A Grigore Ghica Vodă Str., 700487 Iasi, Romania

**Keywords:** eugenol, copaiba essential oil, skin, keratinocytes, biocompatibility

## Abstract

The present study was focused on the preparation, characterization and application onto cotton fabrics of different topical oil-in-water emulsions based on chitosan, eugenol and copaiba essential oil for potential topical applications. Different amounts of copaiba essential oil (oil phases) and eugenol were used, while the water phase consisted of hamamelis water. The designed formulations were evaluated via optical microscopy and rheological parameters assessment. The textile materials treated with the developed emulsions were analyzed in terms of antibacterial efficiency and in vitro and in vivo biocompatibility. The rheological measurements have shown that the emulsions’ stability was dependent on their viscosity and structure of the colloidal systems. The emulsions remained stable at temperatures equal to or below 35 °C, but an increase in temperature led to droplet flocculation and creaming. The emulsion-treated textiles exhibited antibacterial activity against *Escherichia coli* and *Staphylococcus aureus*, and in vivo biocompatibility on the skin of guinea pigs without sensitization effects. Our study revealed that eugenol and copaiba essential oil-based emulsions loaded on cotton textile materials could be promising candidates for developing skin-friendly textiles designed for different topical applications.

## 1. Introduction

Essential oils have been gaining increasing interest in various areas [1,2,3,4,5,6]. Essential oils, also called volatile natural mixtures, are secondary metabolites of plants with antimicrobial [7], antiviral [8], antioxidant [9], anti-inflammatory [10], anti-allergic [11] and insecticidal [12] properties. As natural products, they are not harmful to human body, do not create resistant microorganisms and are environmentally friendly, non-toxic, non-mutagenic and non-teratogenic. They have beneficial effects on the human body and possess environmentally friendly characteristics. They also have a relevant biological activity, being used in the medical field thanks to their biocidal (bactericidal, virucidal and fungicidal) and medicinal activity [13,14].

Several studies have highlighted the antimicrobial efficacy of essential oils, even against multi-resistant bacteria [15,16]. Among the essential oils, copaiba essential oil is an attractive natural alternative to synthetic pharmacological agents, which are frequently linked to severe side effects. Copaiba essential oil is extensively utilized across various industries, including cosmetics, food and wellness, for its exceptional versatility and benefits [17,18]. Copaiba essential oil is well known for its pleasant scent and skin-softening qualities, making it a common ingredient in many cosmetic products. Because it is safe, the U.S. Food and Drug Administration has officially approved using copaiba essential oil as a flavoring in foods and beverages. Copaiba essential oil is a highly versatile concentrated liquid extracted through steam distillation of the oleoresin of the copaiba tree. It contains a complex mixture of terpenes, including β-caryophyllene, a major terpene found in copaiba essential oil that exhibited a wide range of biological activities [19,20].

Eugenol, also known as 4-allyl-2-methoxyphenol, is a naturally occurring phenolic compound that is extensively utilized in the cosmetics, food, pharmaceutical and active packaging industries due to its powerful antimicrobial and antioxidant properties [21,22,23]. Plant-derived bioactive compounds, like essential oils and eugenol, are recognized for their instability and high volatility. These compounds are particularly sensitive to light, oxygen and heat at every stage of processing, utilization and storage [24]. Hence, they can be encapsulated to enhance protection and functionality.

Encapsulation has gained popularity over recent decades as a means of entrapping bioactive principles, owing to their numerous advantages: improved stability, controlled release, protection against oxidation, reduced toxic side effects and so on [25].

The main objective of this study was to develop novel biocompatible textile materials with antibacterial properties by applying different emulsions based on eugenol and copaiba essential oil. Firstly, we investigated the rheological properties of the designed formulations, and since the bioactive compounds used in the production of the emulsions have a wide range of biological activities, we evaluated the biocompatibility of the textile materials treated with emulsions stabilized with chitosan for potential topical applications.

## 2. Materials and Methods

### 2.1. Materials

Copaiba essential oil (*Copaifera officinali*s) was purchased from Aromateria, Targu-Mures, Romania, and eugenol (99%), chitosan (low molecular weight and degree of deacetylation of 0.85) and Tween 80 were supplied by Sigma–Aldrich, Darmstadt, Germany. Glycerol from Honeywell, Charlotte, NC, USA, was used as a solubilizing agent, and the hamamelis water from Mayam (Elemental SRL, Oradea, Romania) formed the water phase of the emulsions. For laboratory experiments, 100% raw woven cotton fabric with a plain weave and a weight of 168 g/m^2^ was used. The textile material was manufactured in the Weaving Pilot Station of INCDTP Bucharest, Romania. For preliminary preparation of the textile materials, various chemical reagents and finishing auxiliaries were used. NaOH, Na_2_CO_3_, Na_3_PO_4_ were obtained from Consors SRL, Bucharest, Romania, and Kemapon PC, Kemapol SR 40 Liq and Kemaxil Liq H_2_O_2_ were purchased from Kem Color S.p.a, Torino, Italy.

### 2.2. Formulation of Oil/Water (O/W) Emulsions

Chitosan-based emulsions loaded with eugenol and copaiba essential oil were prepared using a method consisting of successive stages, i.e., oil-in-water (*o*/*w*) emulsion, as we described in our previous study [26]. Chitosan solution (3% *w*/*v*) was prepared by stirring chitosan in a 2% *v*/*v* aqueous acetic acid solution at room temperature overnight. Over the prepared chitosan solution, the distilled water, glycerol and 30% (*v*/*v*) Tween 80 were separately added dropwise under vigorous magnetic stirring using a stirring rate of 700 rpm, for 10 min during each stage. After complete homogenization, the copaiba essential oil and eugenol were slowly dropped into the stirred mixture, which was maintained under agitation at room temperature for 10 min at each stage. For all five experimental variants, the ratio between eugenol and copaiba essential oil was kept constant (1:1) (Table 1). All emulsions were prepared at room temperature (22 ± 0.1 °C).

### 2.3. Preliminary Treatments of Textile Materials

The hydrophilicity of textile materials needed for further functionalization was achieved in two steps, as previously described in detail in [27], which included a hot alkaline treatment at 95 °C for 90 min in a bath containing NaOH, Na_2_CO_3_, Na_3_PO_4_, Kemapon PC and Sequion, followed by a bleaching at 98 °C for 60 min in a mix containing H_2_O_2_, NaOH, Kemaxil and Kemapon PC. Between these steps, several washings were performed (at 80 °C, 60 °C, 40 °C and at room temperature for 10 min), and after the bleaching, the fabrics were rinsed at 90 °C, 60 °C and 40 °C, for 10 min each, and last with cold water. At the end, the fabrics were dried at room temperature.

### 2.4. Immobilization of Emulsions on the Textile Materials

After the preliminary preparation, the textile materials (20 cm × 20 cm) were then treated with obtained emulsions (after 24 h from their preparation) using the padding method until they reached a wet pick-up rate of approximately 85%. A laboratory-scale padder BVHP 2 (Roaches, West Yorkshire, UK) with two rollers was used with the following settings: 2 passes, 1 m/min and with a pressure of 0.74 bar. Following this, the treated textile materials were subjected to a drying process at a temperature of 50 °C for a duration of 4 min. The drying operation was performed on a drying/curing/heat-setting unit, model TFO/S 500 mm (Roaches, West Yorkshire, UK).

### 2.5. Analysis of Emulsions

#### 2.5.1. Optical Microscopy

Optical microscopy was performed using an OLYMPUS optical microscope model BX51 (Olympus, Tokyo, Japan), the images being captured with 100× oil immersion objective by an attached OLYMPUS UC30 camera. The droplet size was quantified based on the optical microscopy images (n = 3 random microscopic fields) with the ImageJ software version 1.54f (ImageJ, NIH, Bethesda, MD, USA).

#### 2.5.2. Creaming Index (CI)

The creaming index of emulsions was determined at 0, 8, 24, 48 and 72 h post-preparation (the storage being made in sealed 10 mL bottles at 22 ± 1 °C), as previously described [28]. The measurement was performed in triplicate and the results were expressed as average values.

#### 2.5.3. Conductometric Analysis

The conductivity of emulsions was assessed directly at 22 ± 0.1 °C using the C1020 Consort conductometer (Merelbeke, Belgium) with SP10T Consort electrode.

#### 2.5.4. Rheology Measurement

The rheological measurements were performed on the Physica MCR 501 rheometer (Anton Paar, Graz, Austria) with a 50 mm diameter plate–plate measuring system for amplitude sweep, frequency sweep, temperature tests, time tests and flow tests. The amplitude sweep was performed with a constant frequency of 10 rad/s and the amplitude ranged from 0.01 to 100%. A constant strain within the linear viscoelastic range (LVR) was maintained during the frequency sweep with a frequency range from 0.1 to 500 rad/s. Experiments were performed to investigate the influence of temperature (20–50 °C) and time at 25 °C on constant amplitude (1%) and constant frequency (1 Hz). Flow curves were obtained in the shear rate domain 0.01–1000 s^−1^.

### 2.6. Characterization of the Functionalized Textile Materials

#### 2.6.1. Measurement of Antibacterial Activity

The antibacterial activity was qualitatively analyzed by using the ISO 20645:2004 [29] standard method, as described in our previous studies and briefly described in the following one. The assessment of antibacterial activity involved testing the cultures of ATCC 6538 *Staphylococcus aureus* and ATCC 11229 *Escherichia coli* strains. For the testing, the treated textile materials were cut into 20 mm diameter circular shapes and placed in the center of Petri plates. Then, a two-layer culture medium was poured into the plates, with the lower layer containing bacteria-free culture medium (150 mL) and the upper layer inoculated with 5 × 10^8^ CFU/mL of the test bacteria, and then were subjected to an incubation period of 48 h at 37 °C. Finally, the samples were analyzed based on the absence or presence of bacterial growth in the contact zone between the agar and the sample and on the eventual appearance of an inhibition zone. The results were determined by measuring the diameter of the inhibition zone in mm. For the testing of antibacterial activity, the untreated fabrics were used as control samples.

#### 2.6.2. In Vitro Cytotoxicity Determination of Fabrics Extracts

To evaluate the biocompatibility of fabrics treated with bioactive polymer systems, human keratinocytes (HaCaT cell line) were cultured in Dulbecco’s Modified Eagle Medium (DMEM, Sigma-Aldrich, St. Louis, MO, USA) with 10% fetal bovine serum (FBS, Gibco, Grand Island, NY, USA) at 37 °C in a 5% CO_2_ humidified atmosphere, according to the method outlined by Chirila et al. [26].

First of all, the extracts from each fabric sample were prepared as described by Fanizza et al. [30]. The small fabric pieces (0.3 cm × 0.3 cm) sterilized under UV light for 72 h were incubated in 1 mL of DMEM with FBS for 24 h under continuous mixing at 240 rpm. Keratinocytes seeded onto 96-well plates at a density of 3 × 10^4^ cells/well were allowed to adhere overnight, and then exposed to 100 µL of fabric extracts per well for 24 h.

Firstly, the membrane integrity after incubation with fabric extracts was evaluated throughout lactate dehydrogenase release. Cell culture supernatants were incubated in the dark for 30 min at room temperature with the dye and catalyst from the Cytotoxicity Detection Kit (Roche, Mannheim, Germany), performing an absorbance read at 490 nm using a FlexStation 3 (Molecular Devices, San Jose, CA, USA). Secondly, the nitric oxide (NO) amount was measured based on a Griess colorimetric test by reading the optical density of the medium mixed with Griess reagent at 550 nm. Thirdly, the measurement of cellular viability was performed using the MTT [3-(4,5-dimethylthiazol-2-yl)-2,5-diphenyltetrazolium] assay, which assessed the reduction of MTT reagent to a purple formazan. Statistical analysis was performed using one-way analysis of variance (ANOVA) followed by Bonferroni post-test in order to correct for the multiple comparisons performed, the Bonferroni-corrected significance level α being set at 0.01 (0.05/5, as we analyzed five different comparisons).

To observe the amount of viable and dead cells after 24 h exposure to the fabrics’ extracts, the LIVE/DEAD^TM^ assay kit (Invitrogen, Thermo Fisher Scientific, Waltham, MA, USA) was used in accordance with the guidelines of the manufacturer. Images were captured with Olympus IX71 (Olympus, Tokyo, Japan).

#### 2.6.3. In Vivo Dermal Test of Emulsion-Treated Fabrics

The in vivo biocompatibility of the cotton fabrics treated with emulsions (Table 1) was assessed within the National Institute for Chemical-Pharmaceutical Research & Development (INCDCF) by testing the sensitizing potential according to the method of the occlusive dressing test (Buehler test) from the ISO10993-10:2021 standard [31]. The activity of use and maintenance of laboratory animals within the INCDCF vivarium was carried out on the basis of the authorization issued by the National Veterinary Sanitary and Food Safety Authority (ANSVSA), no. 353/24.04.2017, in compliance with the provisions and regulations in the field issued by the Federation of European Laboratory Animal Science Associations (FELASA) and taken over by Romanian Association for Laboratory Animal Science (ARSAL), according to Law no. 43/2014, Directive 2010/63/EU. In order to carry out the tests, the project had the Ethical Committee approval, registered at Veterinary Sanitary and Food Safety Directorate (DSVSA) Bucharest (Romania), no. 9035/28 September 2022.

Fifty-five healthy albino Dunkin–Hartley guinea pigs with a minimum weight of 200–300 g were used, provided by the animal facility “Animaleria SPF” (Baneasa Station, Cantacuzino Institute, Bucharest, Romania). The animals were distributed 10 per experimental lot (R1CE, R2CE, R3CE, R4CE and R5CE) and 5 for the control represented by a dressing with distilled water. The number of animals was chosen this way as it was considered that the material does not contain substances with possible irritating action.

The animals were checked at the reception and kept for a period of 7 days for acclimatization in the INCDCF vivarium, observing clinical condition, behavior and food consumption. The animals were housed in the experimental room with temperature conditions of 22 ± 2 °C and relative humidity of 50–60%, artificial lighting and alternating 12 h of light/12 h of darkness, placed in transparent polycarbonate cages corresponding to the size of the batches, covered with a grid made of stainless steel and provided with an area for food in the form of granules and water. The animals received standardized food and water ad libitum.

A 25 cm^2^ fur area from the dorsal region of the animals subjected to the experiment was removed by shaving approximately 24 h before the start of the test. During the study, it was necessary to repeat the fur removal at an interval of approximately 7 days.

The test samples (R1CE, R2CE, R3CE, R4CE and R5CE) cut in surfaces of 2 cm × 3 cm were placed in intimate contact with the moistened skin and covered with immobilized dressing with semi-occlusive adhesive tape for approximately 6 h. After this period, the occlusive systems were removed. The procedure of applying the samples to be tested was repeated 3 consecutive days out of the week for a period of 3 weeks. The control lot was subjected to similar maneuvers, using only dressing and distilled water.

Fourteen days after the last “induction” application, the batches of animals were exposed to the “challenge” step. The test samples were applied to the previously depilated skin, being kept in contact with the skin for 6 h both in the tested and control groups. After this interval, the occlusive dressing was removed and the surface gently washed with distilled water. The observation of the animals was carried out in an environment with natural lighting to detect the reactions present at the level of the exposed skin. An observer not involved in the experiment was used to reduce subjective evaluation errors.

## 3. Results and Discussion

### 3.1. Morphological Analysis of Emulsions

**Figure 1:**
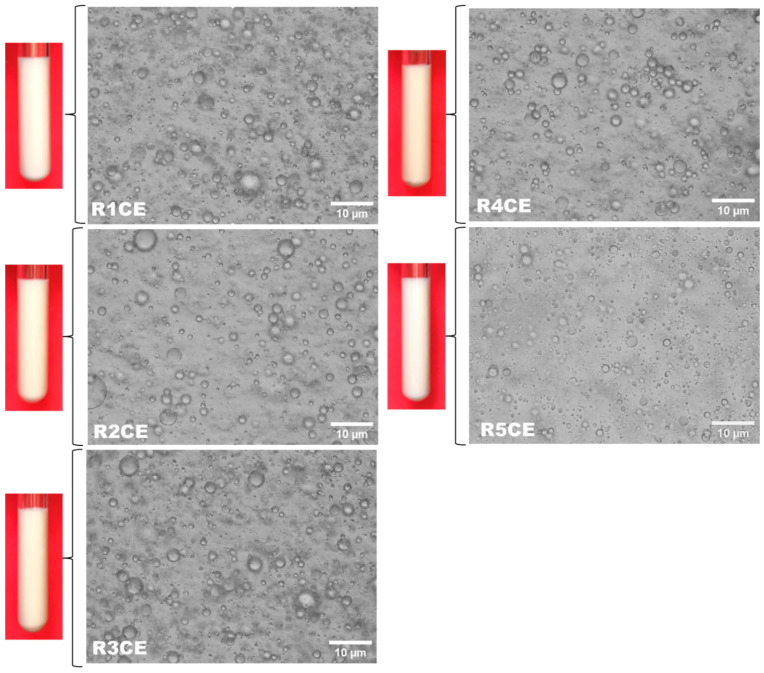
Digital photographs of tubes with prepared emulsion and optical microscopy images (100× oil immersion objective) showing the droplets of the formulated emulsions. The images were taken 24 h after the emulsions were prepared.

Figure 1 depicts the appearance of the five emulsions developed in this study. Chitosan-based emulsions loaded with eugenol and copaiba essential oil showed a whitish color and homogeneity, indicating that all the oil was emulsified during the preparation stage. In addition, the emulsions display a pleasant, creamy, moisturized appearance and the specific fragrance of the bioactive principles used in the composition. The obtaining of skin-friendly systems containing sustainable and biodegradable products can be extremely challenging due to instability constraints. Digital photographs and photomicrographs of emulsions show no phase separation after 24 h from their preparation. Also, optical microscopy images show that the droplets of the obtained emulsions were all intact and spherical, with a compact network structure which hinders the collision of the droplets, preventing their coalescence and Ostwald ripening, and hence enhances the emulsion stability. In addition, the droplet diameter (Table 1) was almost uniform among samples, ranging between 2 and 3 µm.

### 3.2. Physicochemical and Stability Analysis of Emulsions

Formulating emulsions with sustainable and biodegradable materials is complex because these materials often pose challenges to maintaining stability. Achieving the right balance of ingredients to ensure the emulsion remains stable and environmentally friendly requires careful consideration and expertise. Particle migration (creaming, sedimentation) and particle size variation (flocculation, coalescence) are important factors affecting emulsion stability. Creaming occurs when the density of the disperse phase is lower than that of the medium due to gravity, resulting in a 0% creaming index for an ideal emulsion. A higher creaming index indicates more aggregation and larger flocs. The emulsions with a low creaming index exhibit a good creaming behavior and emulsion stability.

The stability of emulsions relies on the type and amount of surfactants contained. It is well known that Tween 80 has usually been used as a surfactant to stabilize oil-in-water emulsion [32]. Tween 80 surfactant consists of fatty acid esters of polyethylene-glycosylated sorbitol featuring low critical micellar concentrations (CMC = 0.012 mM) and hydrophilic–lipophilic balance values exceeding 14. Each synthesized emulsion contained an equivalent quantity of Tween 80, a component essential for ensuring the fine dispersion of oil particles by decreasing the surface tension at the oil/water interface, which helps to keep the emulsion stable. In contrast, the oil-phase concentration varied, ranging from 1% (*v*/*v*) to 3% (*v*/*v*).

**Figure 2:**
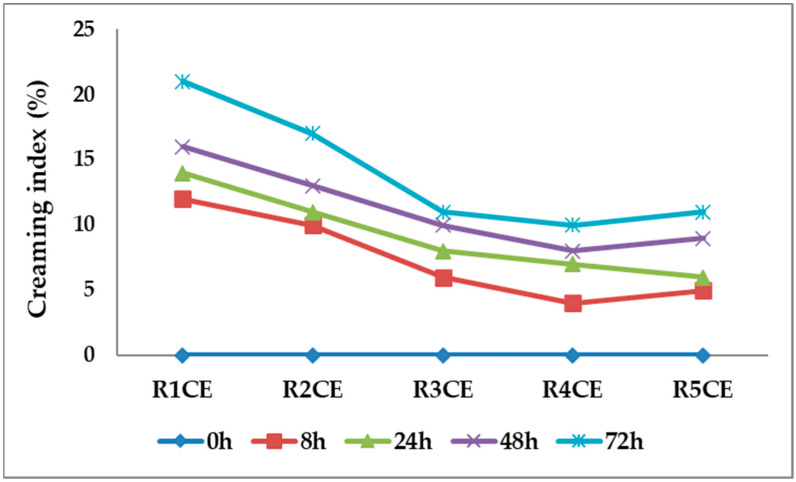
Creaming index values over time obtained for the emulsions containing copaiba essential oil and eugenol.

The emulsions’ stability was assessed by determining the creaming index (CI%), shown in Figure 2. No phase separation was observed in the freshly prepared formulations (0 h). Further, the unchanged CI% after storage indicated excellent emulsion stability as it was observed also for other types of emulsions [33]. After a 72 h storage period, there was a slightly visible sedimentation, which resulted in the formation of a layer of cream at the bottom of the glass bottles due to the density difference in the two phases. The findings indicate that emulsion R4CE demonstrated the highest stability (which contained the higher content of chitosan and lower content of bioactive principles (copaiba essential oil and eugenol)), while emulsion R1CE (which contained the least amount of chitosan) exhibited lower stability.

The electrical conductivity values recorded for the synthesized emulsions are presented in Table 2. Emulsions can be classified as either oil-in-water (O/W) or water-in-oil (W/O) based on their composition. The first phase mentioned represents the dispersed phase, while the second represents the continuous phase. The most conventional methods used for the detection of the type of emulsion include conductivity measurement [34] and microscopy [35].

Within our study, conductivity measurements were carried out to detect the type of the prepared emulsions and also to evaluate the stability right after preparation (0 h) and after 4 h of storage at room temperature (22 ± 1 °C). For the oil-in-water (O/W) emulsion, water is the continuous phase which is conducting and oil is the dispersed phase which is non-conducting. It should be noted that any significant decrease in conductivity over time indicates weak stability of size and implies the loss of integrity, eventually leading to coalescence. In all cases, the measured values were below 500 μS/cm, confirming that the prepared emulsions were of the oil-in-water type [36]. The emulsion with the smallest amount of the active principle (copaiba essential oil and eugenol, sample R4CE) exhibited the highest electrical conductivity value. On the other hand, emulsions containing the same amount of active principles (samples R1CE, R2CE and R3CE) displayed varying reduced values based on the water content. As the water content decreased, the conductivity values also decreased. Furthermore, no significant changes in conductivity values were observed after 4 h of storage.

### 3.3. Rheological Analysis of Emulsions

The rheological behavior of emulsions is a consequence of microstructural modifications [37,38]. Chitosan (CS) has been identified as a natural emulsifying agent and stabilizer due to its hydrophilic character, significant molecular weight and steric stabilization properties [39]. CS displays weak surface activities due to its hydrophilic character, which can stabilize emulsions by generating an enlarged network and boosting the viscosity of the continuous phase [40,41].

The rheological properties of the emulsions were studied using various tests (amplitude sweep, frequency sweep, flow curves, time and temperature tests).

#### 3.3.1. Amplitude Sweep

**Figure 3:**
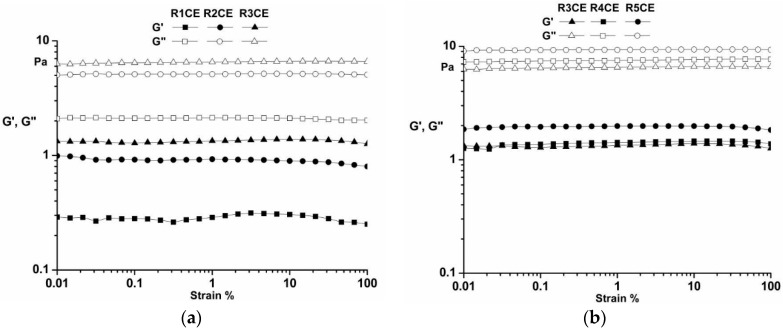
The amplitude sweep for the emulsions: (**a**) R1CE, R2CE and R3CE; (**b**) R3CE, R4CE and R5CE.

The rheological properties of emulsions depend on the system’s components. The amplitude sweep results (Figure 3) illustrate that all samples exhibited stable liquid-like behavior (G″ > G′). The limit of the LVR was determined to be between 1 and 10%, indicating a broad stability range for the emulsions. The dynamic moduli remained unchanged with shear strain, suggesting a wide LVR and good shear resistance in the samples. The properties were evidently influenced by the CS content. The dynamic moduli were observed to increase with the increase in CS concentration in these emulsions, as indicated by the G′ and G′′ values of the samples [40,41,42].

#### 3.3.2. Frequency Sweep

**Figure 4:**
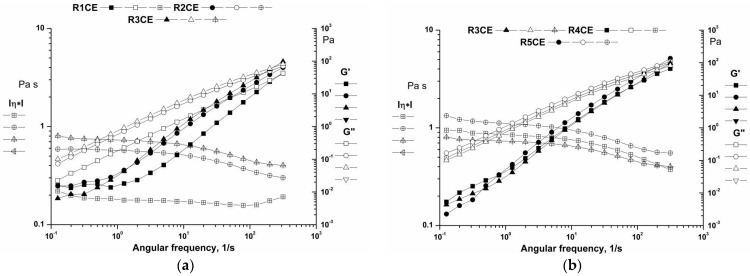
The frequency sweep tests for the emulsions: (**a**) R1CE, R2CE and R3CE; (**b**) R3CE, R4CE and R5CE.

In order to comprehend the structure of the emulsions, angular frequency was used to measure the storage modulus (G′) and loss modulus (G″) (Figure 4). The dynamic moduli G′ < G″ can explain viscoelastic liquid behavior in stable emulsions. Changes in the CS content caused an apparent modification in the mechanical spectra’s shapes. Both moduli were frequency-dependent and showed an increase with frequency. This result is indicative of the appearance of physical entanglements in materials, which may be attributed to the interactions between Tween 80–CS and CS–CS macromolecules [37,40,43]. As the concentration of CS increased, emulsions with slightly higher viscosity values were obtained. The increased inter-particle interactions at higher concentrations of CS increased the probability of structural re-organization [44].

#### 3.3.3. Flow Curves

The study of the rheological properties is crucial in assessing the stability of the emulsions [45]. These parameters are fundamental in preparing emulsions that find successful applications in the food, cosmetic and medical industries [42].

**Figure 5:**
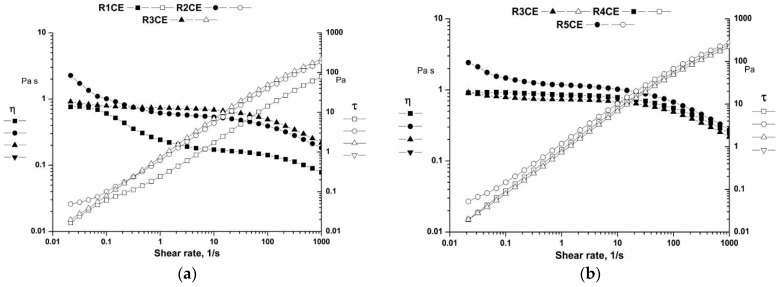
Flow curves for the emulsions: (**a**) R1CE, R2CE and R3CE; (**b**) R3CE, R4CE and R5CE.

Rotational tests were performed at a constant temperature of 25 °C. A shear-thinning behavior was observed for all samples (Figure 5); the viscosity decreased as the shear rate increased [46]. This suggests a reduction in weak bonds (hydrogen bonds) between components, causing the particles to flow in the direction of deformation [47,48]. With an increase in shear rate, all the particle–particle interactions are disturbed, resulting in a decrease in viscosity [49,50,51].

The stability of the emulsions can be enhanced by adding CS. The inclusion of CS heightens the viscosity of the continuous phase, impending the diffusion of droplets and leading to a reduction in the separation rate. The impact of CS on emulsion viscosity was observed to be moderately dependent on the degree of deacetylation, but heavily contingent on its molecular weight and concentration [39,52]. At higher concentrations of CS, the macromolecular chains become increasingly connected, restricting the freedom of movement of the individual chains due to an increased number of entanglements [53]. The addition of CS to the emulsions leads to an increase in viscosity and shear-thinning behavior [40,41,42,43,51].

The Carreau model has been selected to represent the flow behavior of emulsions [37,38,39,54,55,56] because it was found to be effective in representing the flow behavior with higher R^2^ (Table 3) [37,44].

The divergence in fitting parameters is due to the ability of CS to generate emulsions by developing a network in the dispersed phase that can adsorb and entrap the oil. Upon elevating the shear rate, the emulsions exhibited comparable shear-thinning behavior, depicted by the similar p values that denote the decrease in viscosity with increased shear rate. The shear-thinning properties of emulsions typically arise due to droplet collapse and polymer molecule alignment in the continuous phase during shearing [44,57].

#### 3.3.4. Time Tests

**Figure 6:**
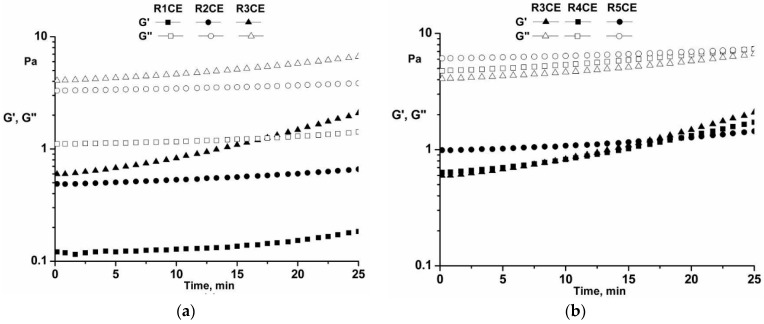
Time test for the emulsions: (**a**) R1CE, R2CE and R3CE; (**b**) R3CE, R4CE and R5CE.

Throughout the experiments (Figure 6), the emulsions displayed a consistently stable structure, as evidenced by the dynamic moduli G′ and G″ maintaining constant values [58]. The adaptable structure of CS, which can change the phase mobility, is a potential contributor to the creation of a stable emulsion [40,52].

#### 3.3.5. Temperature Tests

**Figure 7:**
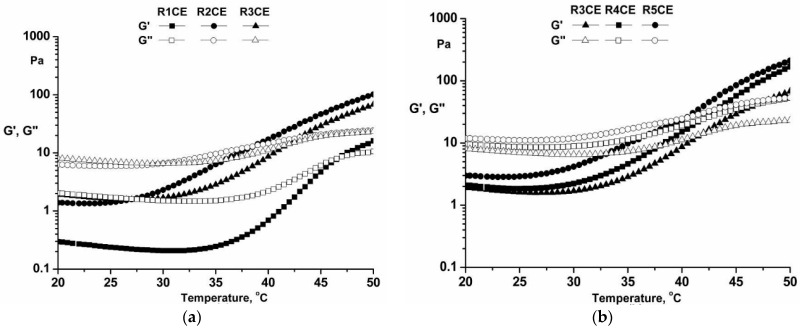
Temperature test for the emulsions: (**a**) R1CEL, R2CE and R3CE; (**b**) R3CE, R4CE and R5CE.

The characteristics of the emulsions were examined using temperature sweep tests (Figure 7), which involved heating the emulsions from 20 to 50 °C at a heating rate of 1 °C/min (frequency f = 1 Hz, strain ɣ = 1%). Above 35 °C, there was a slight rise in the dynamic moduli G′ and G″, which can be attributed to the weak CS molecule bonds interacting. At the critical temperature (37.5 °C), both hydrogen bonds and particle–particle interactions induce emulsion gelation. As temperatures rise above 40 °C, the structure of the emulsions is replaced by the gel structure with dynamic moduli G′ > G′′ [59,60].

According to Figure 5, the emulsions with CS exhibit high dynamic modulus values, indicating that the addition of CS, hydrophobic and hydrogen bonding may strengthen the gel network [61]. Furthermore, the content of CS contributes to the adjustment of heat stability in the emulsions [41].

### 3.4. Analysis of Antibacterial Activity

This study examined the antibacterial properties of textile materials treated with five emulsions based on copaiba essential oil and eugenol against Gram-negative and Gram-positive bacteria, *E. coli* and *S. aureus*, respectively, two prevalent pathogenic bacteria that significantly impact human health. Infections attributed to these bacteria are conventionally addressed with antibiotics. However, in the last decades, a global observation of *E. coli* and *S. aureus* strains exhibiting resistance to numerous antibiotics has been observed. Due to antibiotics’ adverse impact on health and the escalating bacterial resistance, inquiries persist regarding the viability of essential oils as agents possessing antibacterial properties.

**Figure 8:**
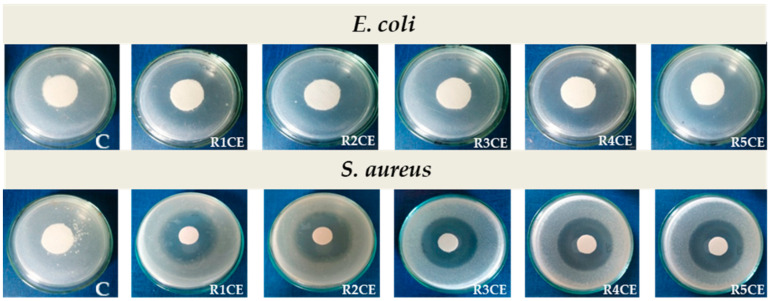
Representative images of Petri dishes showing antibacterial efficiency against *E. coli* and *S. aureus* strains after 48 h in the presence of emulsion-treated fabrics.

The determination of the antibacterial activity for the fabric samples treated with the emulsions developed in this study was qualitatively achieved by using the Agar diffusion plate test according to the ISO 20645:2004 [29] standard with the working methodology previously described, the obtained results being presented in Figure 8 and Table 4.

Based on the data shown in Figure 8, it is evident that samples treated with emulsions containing copaiba essential oil and eugenol demonstrate antibacterial action against both tested bacteria. The untreated samples of cotton did not demonstrate any significant antibacterial activity when tested against *S. aureus* and *E. coli* bacterial strains.

Samples treated with emulsions containing equal amounts of eugenol and copaiba essential oil displayed varying values of inhibition zones. The most significant antibacterial effect was observed for the sample treated with the emulsion containing the highest quantity of chitosan and the lowest amount of hamamelis water (sample R3CE, Table 4). When testing emulsions with the same levels of chitosan (samples R3CE, R4CE and R5CE) but varying amounts of eugenol and copaiba essential oil, we observed slightly higher antibacterial activity in the sample treated with the emulsion containing the highest quantity of bioactive principles (sample R3CE). In the case of the R5CE sample, the diameter of the inhibition zone was slightly lower (Table 4) than for the sample treated with emulsion based on higher amounts of copaiba essential oil and eugenol.

These findings are consistent with the results previously reported by other studies. Strong in vitro antibacterial activity of chitosan films incorporating copaiba oil nanocapsules have been already shown against *Pseudomonas aeruginosa* and *Staphylococcus aureus*. Lipophilic terpenes (β-caryophyllene) are thought to be responsible for this antibacterial activity because they can cause bacterial cell disruption, which can result in intracellular contents leaking out and eventual cell death [62].

### 3.5. Analysis of Fabric Extracts’ Biocompatibility on Human Keratinocytes

**Figure 9:**
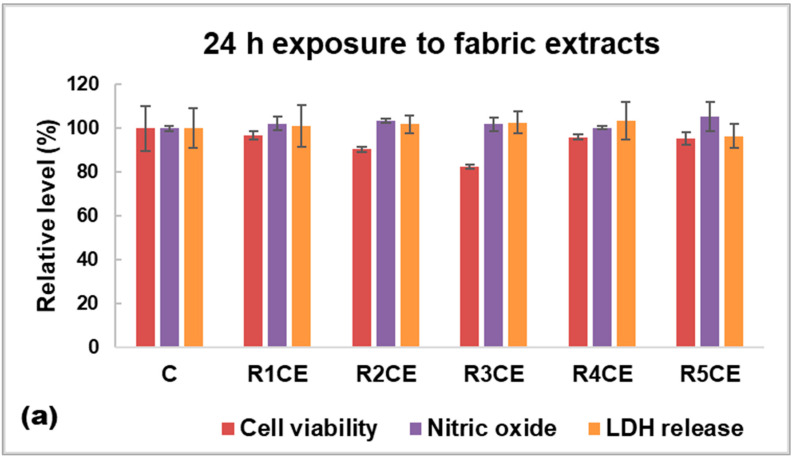

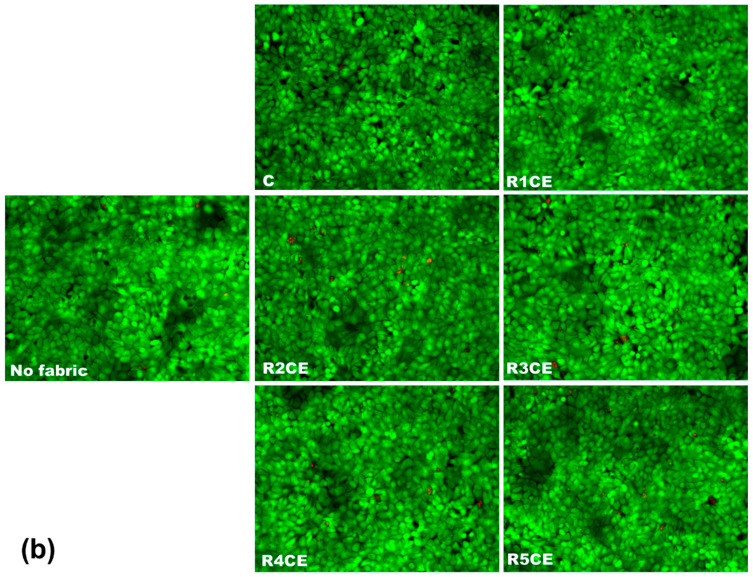
Biocompatibility evaluation of fabric extracts on human keratinocytes (HaCaT cells) after 24 h of incubation via (**a**) quantitative assays, cell viability, nitric oxide level and lactate (LDH) dehydrogenase release tests, and by (**b**) fluorescence staining of live (green labeling with calcein solution) and dead cells (red labeling with propidium iodide). In parallel, cells without any fabrics were analyzed. All images were obtained with objective 20×. Data were calculated as mean ± standard deviation (n = 3) and normalized to HaCaT cells grown in the presence of extracts from untreated fabric (C—control), No significance was obtained after statistical analysis was performed between fabrics’ extracts and untreated fabrics (C).

The biocompatibility evaluation of fabric extracts on HaCaT human keratinocytes demonstrated high overall cell viability across R1CE, R4CE and R5CE samples, showing noticeable reductions compared to the control (untreated fabric) for R2CE and R3CE (Figure 9a). The explanation could be found in the composition of each emulsion (Table 1), high levels of eugenol and copaiba essential oil combined with an increased amount of chitosan being inductors of cell death, decreasing the number of viable cells by 10% and 17% from control one. However, there were no measured significant differences between the samples and control in the case of nitric oxide level and LDH release (Figure 9a). These findings suggest that the components of the emulsion were not triggers of inflammation and lesions in cell membranes.

Furthermore, these results were confirmed by the fluorescence staining of live and dead cells following exposure to fabric extracts (Figure 9b). More dead cells can be observed in the cases of the R2CE and R3CE samples compared to the others, indicating their slightly cytotoxic effect on human keratinocytes.

Previous research showed that copaiba oil did not induce hemolysis in human erythrocytes or cytotoxicity and genotoxicity in A549 cells, indicating that this essential oil is biocompatible and safe to use at concentrations between 50 and 200 μg/mL [63]. Furthermore, Nigro et al. [64] used the MTT assay to assess the cytotoxicity of copaiba oil nano-emulsion on fibroblasts and keratinocytes and discovered a concentration-dependent reduction (up to 30%) effect in mitochondrial enzyme activity in both cell types, for concentrations from 300 μg/mL to 10 mg/mL. Another study performed by de Araújo Lopes et al. [65] proved that nanoencapsulation of eugenol, a primary bioactive monoterpene compound of essential oils, reduced its cytotoxicity on keratinocytes and improved its anti-inflammatory effects in mice, which is consistent with our findings.

### 3.6. In Vivo Evaluation of Emulsion-Treated Fabrics

The recording of skin reactions was performed approximately 1 h after the removal of the occlusive bandages. The results were evaluated and scored according to the Magnusson and Klingman scale, the observations being taken at intervals of 2, 4, 6, 24 and 48 h.

**Figure 10:**
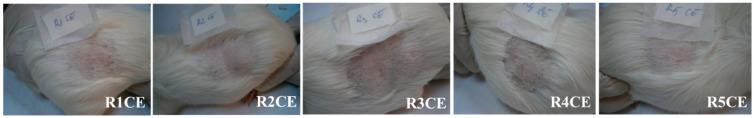
Representative images of skin areas exposed to emulsion-treated fabrics after 48 h from the last “challenge” step of the occlusive dressing test.

Reactions of intensity below 1 according to the Magnusson and Klingman scale were reported, which corresponded to no visual perceptible changes (Figure 10), both in the batches tested with samples R1CE, R2CE, R3CE, R4CE and R5CE, as well as in the control ones during and after the “challenge” period. These findings lead to the conclusion that these textile materials, functionalized with polymer systems in the form of emulsions for skin applications, do not present sensitizing potential on guinea pigs under the conditions of this test performed according to the recommendations of the ISO10993 standard [31] and the rules of good laboratory practice.

## 4. Conclusions

Textile materials with biocompatibility and antimicrobial properties were obtained by applying emulsions containing eugenol and copaiba essential oil on cotton fabrics. Five emulsion formulations containing chitosan (as a polymeric matrix), Tween 80 (as a synthetic emulsifier), glycerol (as a solubilizing agent), hamamelis water (as water phase) and eugenol and copaiba essential oil (oil phase) were designed and characterized. The emulsions displayed a higher loss modulus than storage modulus, showing their high viscosity and suggesting that the stability of the emulsions is dependent on both their viscosity and CS structure. As CS concentration increased, a more CS-dependent network was formed, thereby improving the physical stability of the emulsions by delaying the onset of oil droplet flocculation and creaming. The viscosity of the emulsions was enhanced as the concentration of CS increased, and a shear thinning behavior was noted due to increased entanglement between the macromolecular chains. The antibacterial activity against *S. aureus* and *E. coli* and good biocompatibility on animal skin of these emulsion-treated fabrics highlighted their benefits for skin applications.

## Figures and Tables

**Table 1:** Composition of emulsions expressed as percentage of total (100%). The initial concentrations of chitosan and Tween 80 were 3% and 30% (*v*/*v*), respectively. Droplet diameter was expressed as mean ± standard deviation (n = 3 random fields).

Code	Chitosan	Tween 80	Glycerol	Hamamelis Water	Eugenol	Copaiba Essential Oil	Droplet Diameter (µm)
R1CE	30	1.67	9	53.33	3	3	2.26 ± 0.56
R2CE	40	1.67	9	43.33	3	3	2.38 ± 0.92
R3CE	50	1.67	9	33.33	3	3	2.61 ± 0.77
R4CE	50	1.67	9	37.33	1	1	2.19 ± 0.68
R5CE	50	1.67	9	35.33	2	2	2.15 ± 0.54

**Table 2:** Conductivity values assessed for the developed emulsions right after preparation (0 h) and after 4 h of storage at room temperature.

Parameters	Emulsions									
	R1CE		R2CE		R3CE		R4CE		R5CE	
Time of Storage	0 h	4 h	0 h	4 h	0 h	4 h	0 h	4 h	0 h	4 h
Conductivity (μS/cm)	356.7	353.9	340.2	339.1	331.6	329.4	373.6	372.5	345.2	344.3

**Table 3:** Fitting parameters of the Carreau model to experimental data of emulsions. The results are presented as mean ± standard deviation (n = 2).

Main Fitting Parameters	Emulsions				
	R1CE	R2CE	R3CE	R4CE	R5CE
η_0_ (Pa·s)	0.2408	0.5686	0.7613	0.8692	1.106
η_∞_·10^8^ (Pa·s)	93.3	2.18	2.59	2.93	3.27
p	0.068	0.149	0.152	0.151	0.175
R^2^	0.942	0.979	0.936	0.972	0.986
Standard deviation	±0.06	±0.02	±0.04	±0.03	±0.03

**Table 4:** Evaluation of the antibacterial activity exerted by the treated fabrics. The results were presented as mean ± standard deviation (n = 2).

	Diameter of Inhibition Zone (mm)					
	C	R1CE	R2CE	R3CE	R4CE	R5CE
*E. coli*	0	9.2 ± 1.194	8.7 ± 0.760	13.1 ± 1.563	7.6 ± 1.207	11.2 ± 1.689
*S. aureus*	0	9.4 ± 0.987	9.3 ± 2.209	12.7 ± 1.232	11.1 ± 1.563	12.4 ± 1.447

## Data Availability

The original contributions presented in the study are included in the article, further inquiries can be directed to the corresponding authors.
